# New species and new record of *Statherotmantis* Diakonoff, 1973 from China (Lepidoptera: Tortricidae: Olethreutinae)

**DOI:** 10.1002/ece3.10906

**Published:** 2024-01-31

**Authors:** Wenqing Jing, Zehua Niu, Shuang Ma, Haili Yu

**Affiliations:** ^1^ Shaanxi Key Laboratory for Animal Conservation Northwest University Xi'an China; ^2^ Key Laboratory of Resource Biology and Biotechnology in Western China (Northwest University) Ministry of Education Xi'an China

**Keywords:** barcodes, China, new species, Olethreutini, *Statherotmantis*

## Abstract

In China, six species of *Statherotmantis* Diakonoff, 1973 were previously recorded. In the present study, four other species were recognized using morphology and DNA barcording analysis. Among these, three of which, *S. miniscula* sp. n., *S. calva* sp. n., and *S. longiuscula* sp. n., are described as new. In addition, one species, *S. laetana* Kuznetzov, 1988, is a new record for China. Adults and genitalia are illustrated, and keys to identify the Chinese species of *Statherotmantis* are provided.

## Graphical abstract

In the present study, three new species and one new record are recognized using morphology and DNA barcording analysis.

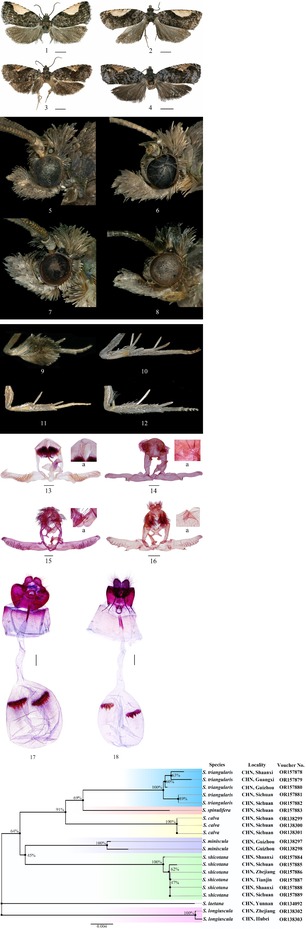

## 1 INTRODUCTION

The genus *Statherotmantis* Diakonoff, 1973 consists of nine species and is distributed in the Palaearctic and Oriental regions (Byun et al., 1998; Diakonoff, 1973; Falkovitsh, 1966; Gilligan et al., 2018; Kawabe et al., 1992; Kuznetsov, 2001; Kuznetzov, 1969, 1988; Li & Yu, 2007; Liu & Li, 2002; Trematerra & Spina, 2009). Prior to the present study, six species of this genus were recorded in China (Kawabe et al., 1992; Li & Yu, 2007; Liu & Li, 2002). During our ongoing studies on the Olethreutini of China, we have discovered three previously undescribed species and one new record species of *Statherotmantis* from southern China.

The identification of *Statherotmantis* species is challenging due to their similar appearance characterized by the dark forewing with a conspicuous triangular or semicircular pale spot over the middle of the costa and bordered with a blackish band. Genitalia examination could discriminate between different species, and DNA barcode surveys are also utilized. In this present study, we present 20 full‐length barcodes (COI gene, 658 bp) representing seven species, including three new species described herein, the newly recorded species, and three previously recorded species (*S. shicotana* Kuznetzov, 1969; *S. triangularis* Li & Yu, 2007 and *S. spinulifera* Li & Yu, 2007). The sequences are available through GenBank (accessed 20 June 2023) and given in Table S1.

## 2 MATERIALS AND METHODS

Specimens were collected using light traps in this study. The detailed collection information of specimens sequenced is shown in Table S1. Genitalia dissection follows the methods described by Li (2002). The specimens were examined using an Olympus SZX71 stereomicroscope, and all images were captured using a digital microscope (VHX‐5000).

Total genomic DNA was extracted using the Genomic DNA Extraction Kit (Tiangen Biotech, Beijing, China) from the legs of dried adult specimens, according to the manufacturer's instructions with Sanger sequencing. The extracted genomic DNA was eluted into 50 μL TE buffer and stored in a freezer at −20°C. The target fragments of COI were amplified following the protocol described by Hebert et al. (2004). The resulting sequences were aligned using ClustalW in MEGA 7 (Kumar et al., 2016), and the genetic distance was analyzed using the Kimura 2‐Parameter model. The sequence data obtained from the specimens have been deposited in GenBank (Accession numbers: OR157878.1–OR157889.1, OR134092.1, OR138297.1–OR138303.1).

The studied specimens, including the types, have been deposited in the Insect Collection of the Northwest University, Xi'an, China (NWU).

## 3 RESULTS

### 3.1 *Statherotmantis* Diakonoff, 1973

*Statherotmantis* Diakonoff, 1973: 288. Type species: *Proschistis pictana* Kuznetzov, 1969.

Key to *Statherotmantis* species from China based on the male genitalia

**Table:** 

1.	Gnathos without median prominence	2
–	Gnathos with a strong median prominence	3
2.	Uncus small, triangular, and naked, less than half of socius in size; gnathos with lateral prominences short, rounded; cucullus slender, less than 1.5 times of aedeagus in width, with ventroproximal base protruding to a basal triangular angle	*S. miniscula* sp. n.
–	Uncus large, subrectangular, with two central hairy areas and naked margins, larger than socius in size; gnathos with lateral prominences long, finger‐like; cucullus more than 1.5 times of aedeagus in width, with ventroproximal base not protruding	*S. shicotana*
3.	Uncus slender, finger‐like; socii with dense fine hairs; gnathos with median and lateral prominences slender, hooked; anellus broad, somewhat cup‐like	*S. laetana*
–	Uncus broad, oval or bilobed; socii with scale‐like spines; gnathos with median and lateral prominences not hooked; anellus a narrow ring	4
4.	Uncus broad, bilobed caudally	5
–	Uncus oval, caudally rounded	7
5.	Gnathos without lateral prominences, median prominence rounded apically	*S. maoerica*
–	Gnathos with distinct lateral prominences, median prominence furcated apically	6
6.	Gnathos with lateral prominences tongue‐like, median prominence T shaped, the stem slender, longer than the apical branches	*S. calva* sp. n.
–	Gnathos with lateral prominences a short angle, median prominence with basal part broad, not constricted, shorter than the apical branches	*S. longiuscula* sp. n.
7.	Gnathos with median prominence truncate apically, distal part not furcated	*S. expansa*
–	Gnathos with median prominence furcated apically	8
8.	Valva with costa possessing a vertical lobe medially	*S. triangularis*
–	Valva without lobes on costa	9
9.	Gnathos with median prominence inverted T shaped apically, lateral prominences broad, tongue‐like, with dense spinules; cucullus elongate triangular	*S. spinulifera*
–	Gnathos with median prominence bifid apically; valva about equal‐width, cucullus not triangular	*S. pictana*

### 3.2 *Statherotmantis laetana* Kuznetzov, 1988

**FIGURES 1–4:**
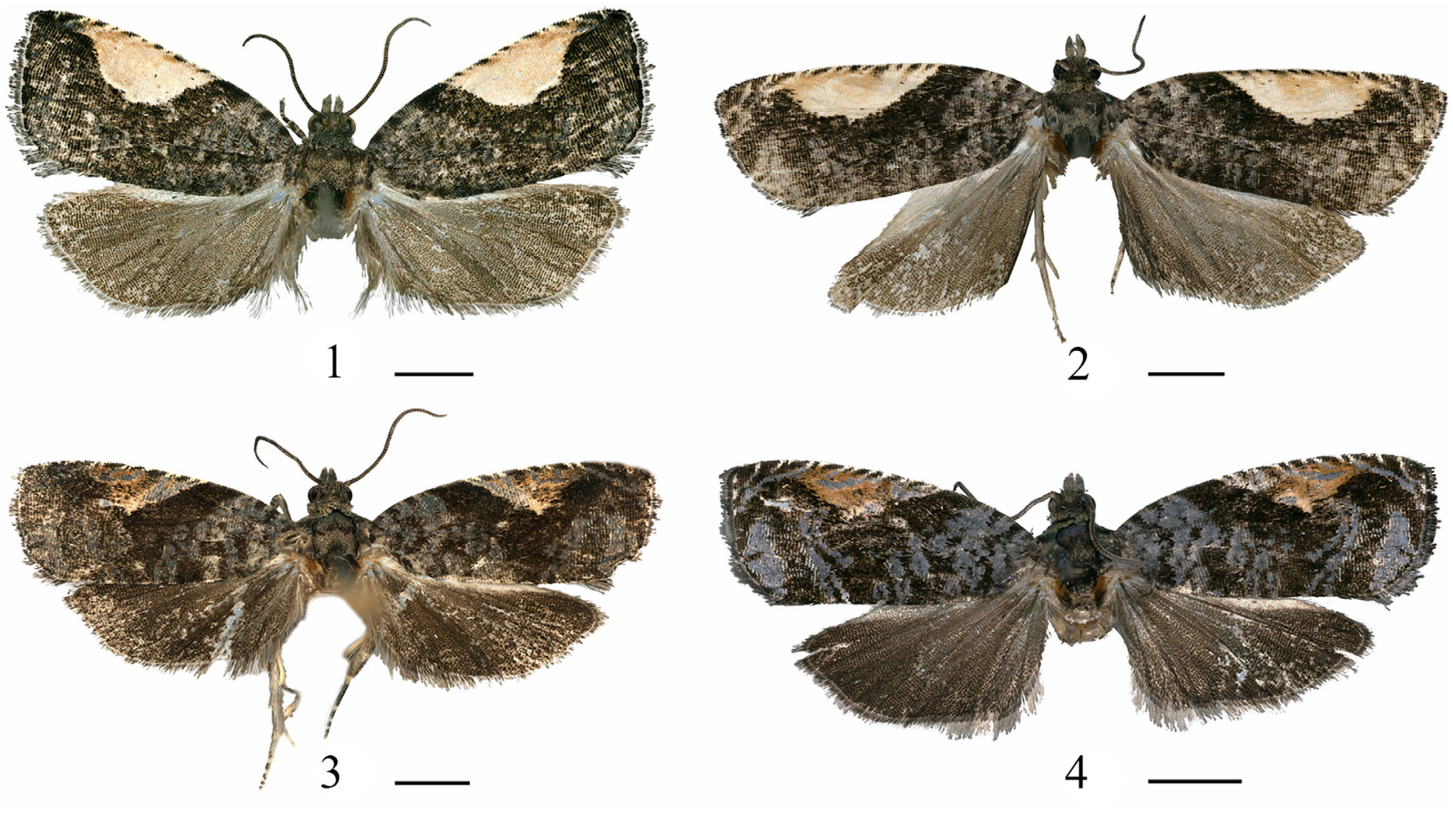
Adults of *Statherotmantis*. (1) *S. laetana*, male; (2) *S. miniscula* sp. nov., male, holotype; (3) *S. calva* sp. nov., male, holotype; (4) *S. longiuscula* sp. nov., male, holotype. Scale bars: 4 mm.

**FIGURES 5–8:**
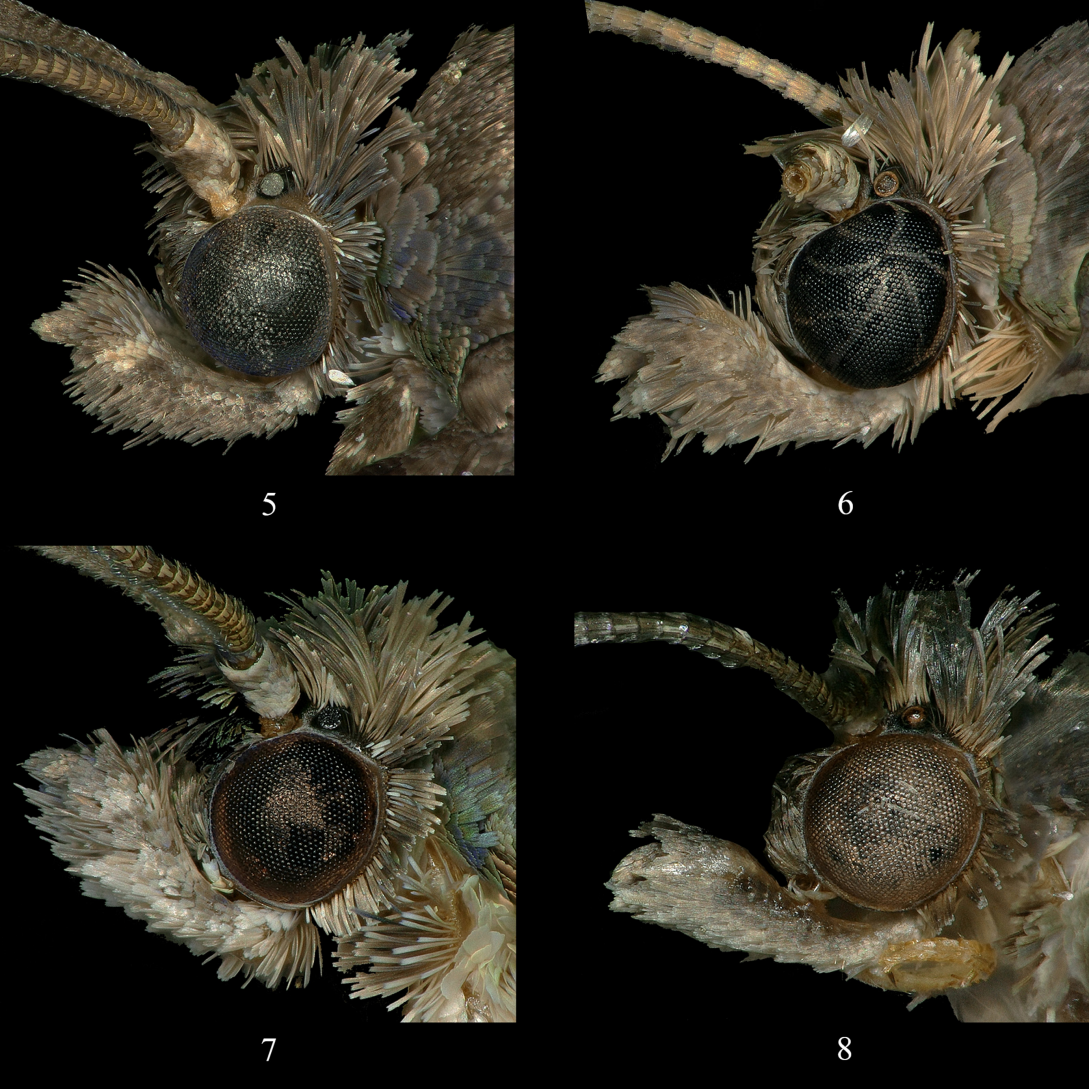
Heads of *Statherotmantis*. (5) *S. laetana*; (6) *S. miniscula* sp. nov., holotype; (7) *S. calva* sp. nov., holotype; (8) *S. longiuscula* sp. nov., holotype.

**FIGURES 9–12:**
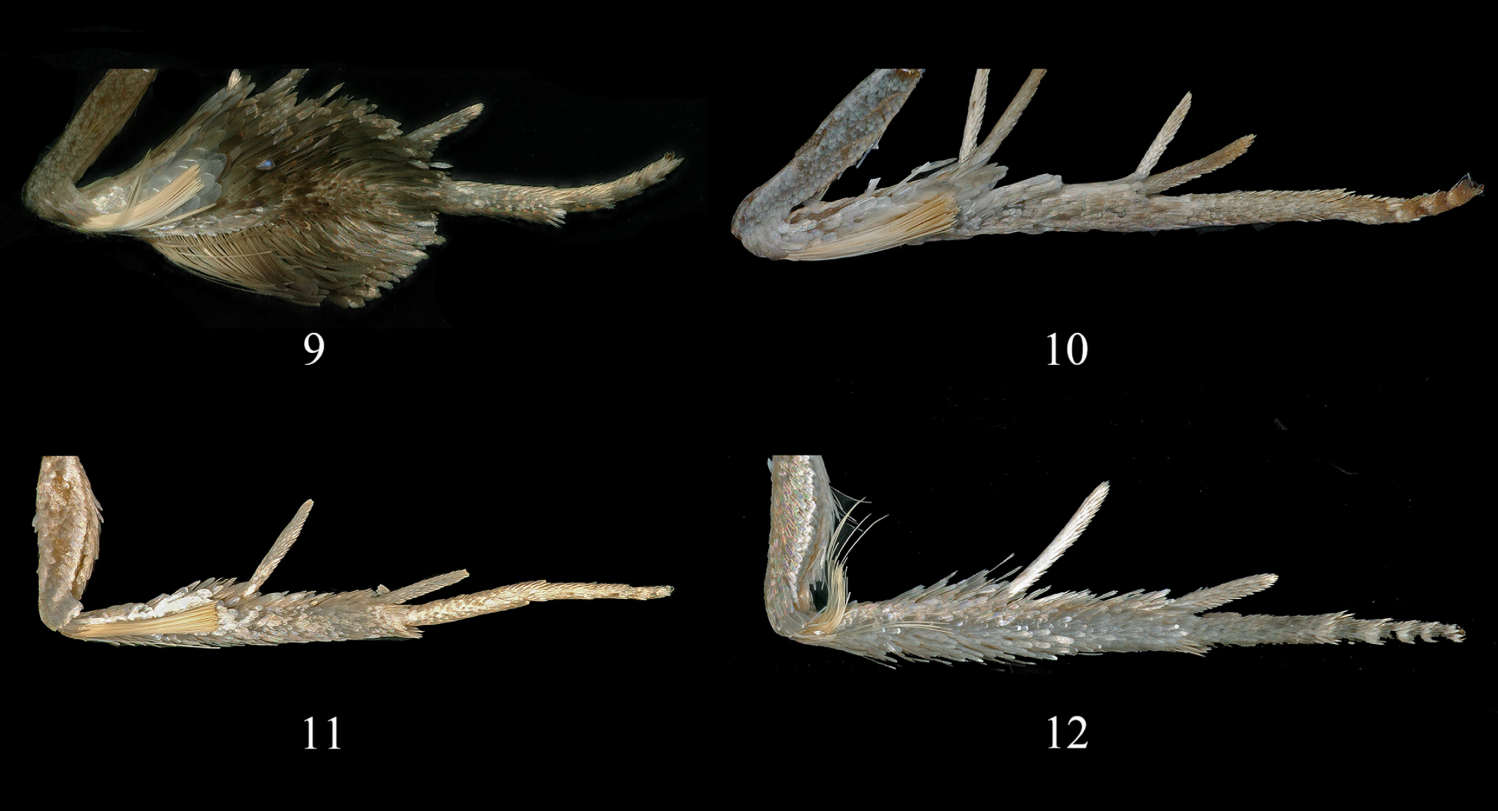
Hind legs of *Statherotmantis*. (9) *S. laetana*; (10) *S. miniscula* sp. nov., holotype; (11) *S. calva* sp. nov., holotype; (12) *S. longiuscula* sp. nov., holotype.

**FIGURES 13–16:**
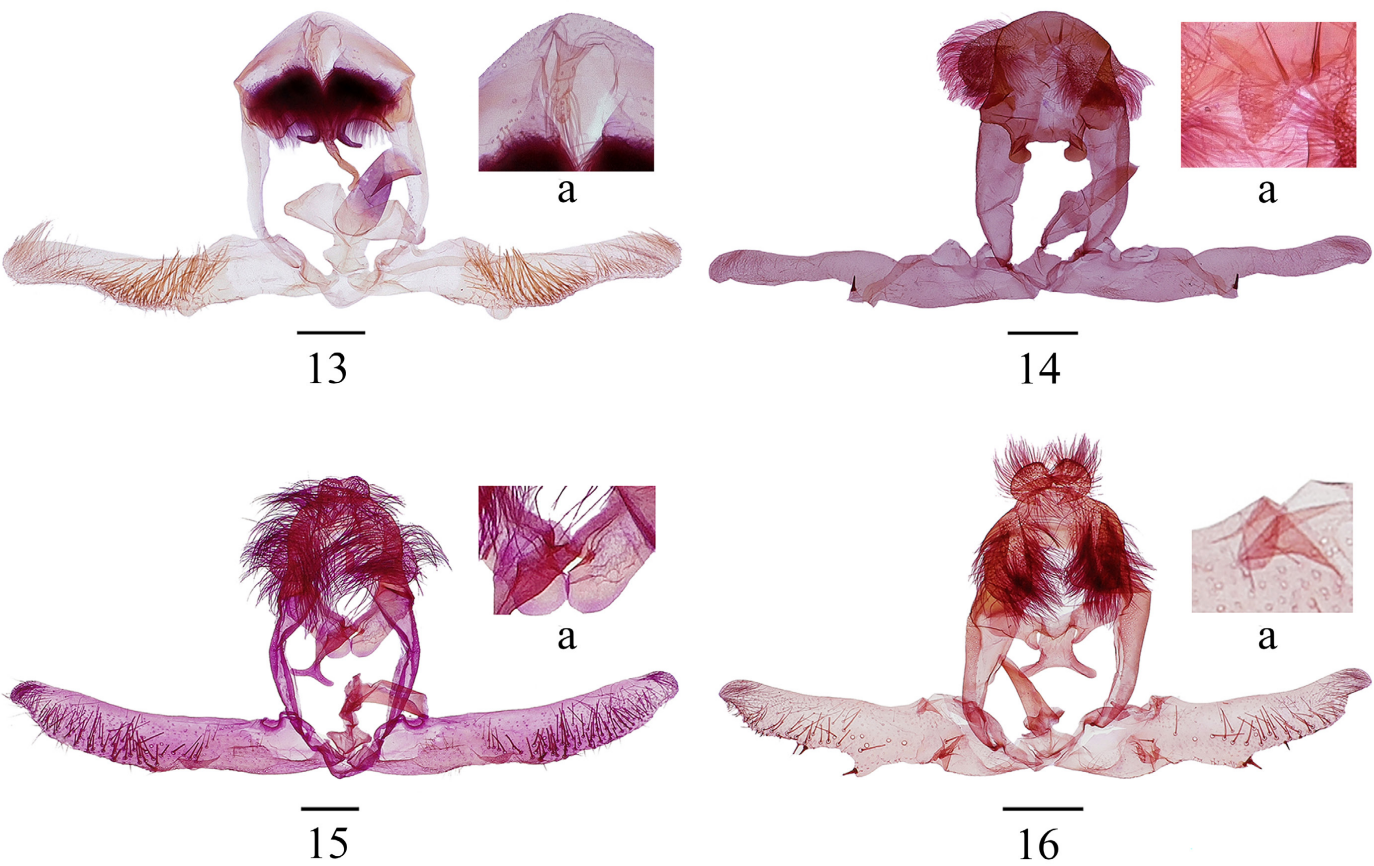
Male genitalia of *Statherotmantis*. (13) *S. laetana*, slide no. LKL13195, (a) uncus; (14) *S. miniscula* sp. nov., holotype, slide no. LYY18506, (a) uncus; (15) *S. calva* sp. nov., holotype, slide no. JWQ21648, (a) naked lateral prominences of gnathos; (16) *S. longiuscula* sp. nov., holotype, slide no. JWQ21617, (a) costal lobe in valva. Scale bars: 1 mm.

*Statherotmantis laetana* Kuznetzov, 1988: 170 (male, Vietnam) (Figures 1, 5, 9 and 13).

Material examined. China, Hainan Prov.: 1♂, Limushan Reserve, 19°17′ N, 109°73′ E, 640 m, 5.V.2014, leg. Tengteng Liu, Wei Guan and Xuemei Hu; 1♂, Bawangling Reserve, 19°12′ N, 109°08′ E, 600 m, 10.VI.2007, leg. Zhiwei Zhang and Weichun Li; Yunnan Prov.: 1♂, Mt. Gaoligong, 27°41′ N, 98°16′ E, 380 m, 1.VI.2017, leg. Kaijian Teng.

This species is in GenBank as GenBank accession number: OR134092.1.

Distribution. Vietnam, China (Hainan, Yunnan).

Note. This species is newly recorded for China. It can be easily distinguished from other species within the genus by the hooked uncus (Figure 13a), the socius with dense fine hairs, the gnathos with long hooked median prominence and short hooked lateral prominences, and the cup‐like anellus in the male genitalia (Figure 13).

### 3.3 *Statherotmantis miniscula* Jing et Yu, sp. nov.

**FIGURES 17–18:**
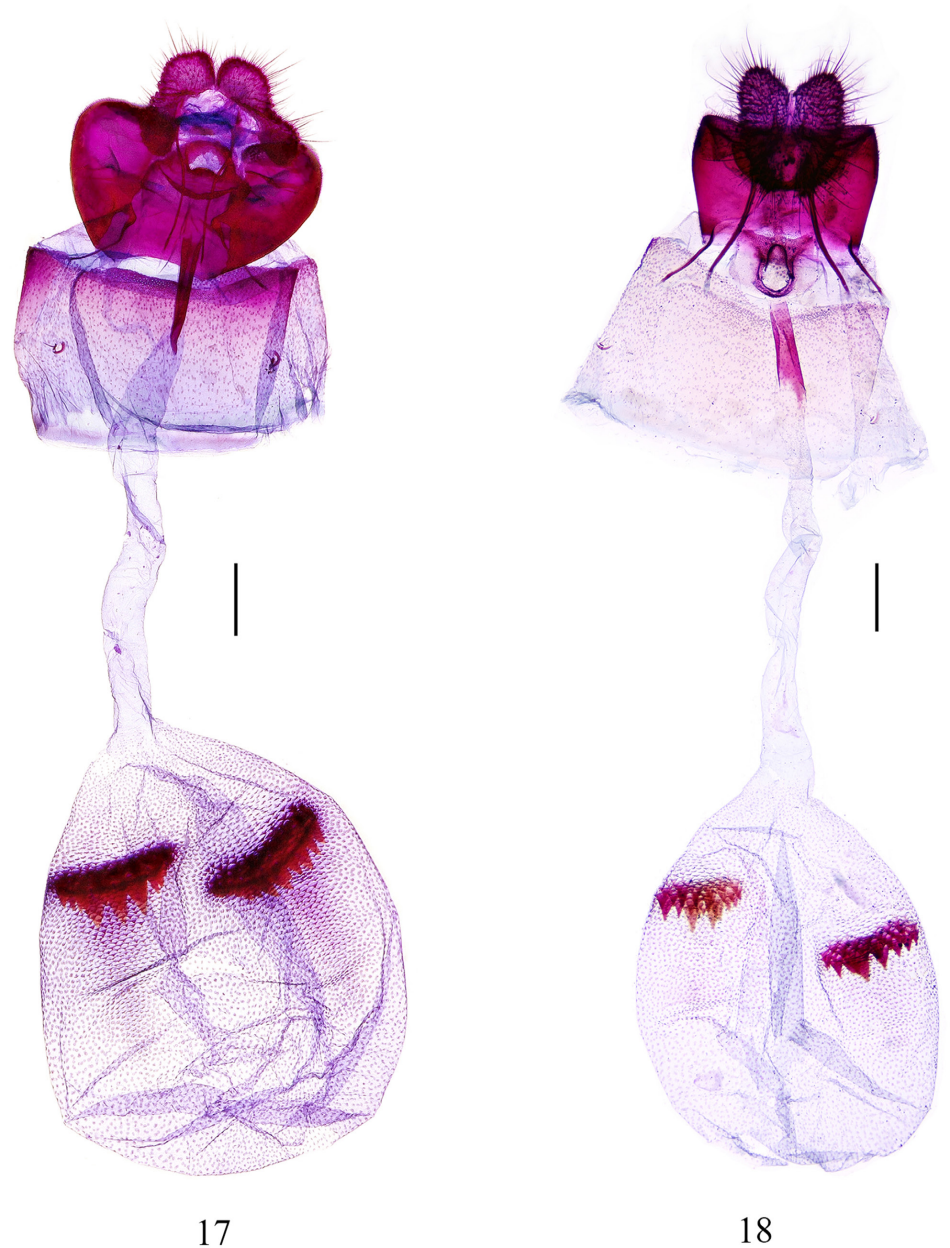
Female genitalia of *Statherotmantis*. (17) *S. miniscula* sp. nov., paratype, slide no. LYY18538; (18) *S. longiuscula* sp. nov., paratype, slide no. SXL20561. Scale bars: 1 mm.

Type material. Holotype: ♂, China, Guangxi Prov.: Mt. Leigong, 26°22′ N, 108°11′ E, 1541 m, 20.VI.2019, leg. Yongyan Li, genitalia slide no. LYY18506. Paratype: 1♂, same data as holotype except 17.VI.2019; 1♀, same data as holotype (Figures 2, 6, 10, 14 and 17).

This species is in GenBank as GenBank accession number: OR138297.1, OR138298.1.

Diagnosis. This newly described species exhibits significant differences in the male genitalia compared with previously described species of *Statherotmantis*. Its uncus is remarkably small, triangular, and pointed at the apex. The valva is slender, and the length of the cucullus is approximately six times the width of its middle. While in other species of the genus the uncus is rounded, bilobed or hooked, and the length of the cucullus is no more than four times the width of its middle. The gnathos of *S. miniscula* resembles that of *S. shicotana* in terms of shape, but it is shorter and the distal part expands to a rounded form. In contrast, the gnathos in *S. shicotana* are finger‐like and doesn't broaden distally. In addition, the distinct male genitalia features, such as the shape of the uncus, valva, and gnathos, clearly differentiate this new species (*S. miniscula*) from other known species, particularly *S. shicotana*.

Description. Adult (Figure 2). Head (Figure 6): ocellus well developed. Vertex is roughly scaled, yellowish brown. Frons dark brown. Antenna dark fuscous, reaching to the middle of forewing. Labial palpus ascending and sinuate, basal segment creamy, median segment pale yellow suffused with pale gray, moderately widened distally, terminal segment porrect, pale gray, and pointed apically.

Thorax: anterior half dark fuscous, posterior half fuscous, without posterior crest. Collar fuscous with pale brown margin. Tegula is dark brown, suffused with brown distally. The hind tibia (Figure 10) in males is not expanded, with a pale yellow hair pencil extending from the base along the inner surface. Forewing length 7.5–8.0 mm; elongate subrectangular, costa slightly curved, distal 4/5 nearly straight, apex blunt, termen oblique; upperside fuscous to blackish fuscous, suffused with pale fuscous, with a large semicircular white mark tinged ochreous centered on costa from about 1/3 to 3/4 length, reaching halfway across the wing, bordered by an indistinct blackish band; pairs of costal strigulae distinctive, nine pairs from the base of costa to R_4_, they are distributed as follows: four pairs (strigulae one to four) between the base of the wing and the point where the Sc meets the costa, two pairs (strigulae five and six) between Sc and R_1_, one pair (strigula seven) between R_1_ and R_2_, one pair (strigula eight) between R_2_ and R_3_, one pair (strigula nine) between R_3_ and R_4_; basal two pairs of strigulae broad, pale fuscous, distal seven pairs white suffused with ochreous, strigula nine represented by a single marking; cilia dark brown, with a white baseline; underside dark brown. Hindwing brown; cilia pale gray; underside grayish white.

Abdomen: pale brown dorsally, pale yellow ventrally. Male genitalia (Figure 14): tegumen broad, moderately high. Uncus (Figure 14a) is small, triangular, and spined. Socius large, dropping, and rounded, densely covered with long hairs. Gnathos without median prominence, lateral prominences constricted basally, short and rounded, naked. Valva slender, basal part slightly broader than the cucullus, the protruding rim of the basal excavation raised from the base of the costa across the cucullus and connected with the projecting ventroproximal angle of the cucullus; sacculus narrow, with sparse short hairs; cucullus very narrow, approximately six times the width of its middle in length, sparsely hairy, ventroproximal base protruding to an acute angle and carrying a strong, short thorn apically. Caulis very short, narrow; anellus broad, more or less cup‐shaped; aedeagus moderate in width and length, slightly narrow distally; cornuti absent. Female genitalia (Figure 17): ovipositor lobes elongate‐ovate. The apophyses anterior is shorter than the apophyses posterior. The 8th tergite is well sclerotized. Sternum 7 with a nearly straight hindmargin. Sterigma is somewhat circular, with a raised central rim encircling the entrance to the ostium, and lower lips (lamella antevaginalis) broad, aciculate. The ostium is somewhat semicircular, flat. Ductus bursae are membranous and smooth, with a long colliculum. Corpus bursae are large, ovate, and densely granulated, with two pectinate signa almost equal in size.

Etymology. The specific name is derived from the Latin *minisculus* (=small), referring to the small uncus in the male genitalia of the species.

### 3.4 *Statherotmantis calva* Jing et Yu, sp. nov.

Type material. Holotype: ♂, China, Sichuan Prov.: Mt. Emei, 29°34′ N, 103°24′ E, 731 m, 19.VII.2021, leg. Ruiqin Dong, genitalia slide no. JWQ21648. Paratype: 13♂, same data as holotype; 3♂, same data as holotype except 20.VII.2021 (Figures 3, 7, 11 and 15).

This species is in GenBank as GenBank accession number: OR138299.1, OR138300.1, OR138301.1.

Diagnosis. This new species mostly resembles *S. spinulifera*, particularly in the male genitalia. However, it can be distinguished by the male genitalia with uncus bilobed, and gnathos with lateral promimences naked; in *S. spinulifera*, the uncus is rounded and the lateral prominences of gnathos are densely spinulose.

Description. Adult (Figure 3). Head (Figure 7): vertex roughly scaled, grayish brown to fouscous. Frons dark brown to fuscous. Antenna dark brown, reaching to the middle of the forewing. Labial palpus sinuate, porrect or ascending, basal segment creamy, median segment pale brown or grayish brown, suffused with fuscous laterally, distally moderately widened, terminal segment porrect, gray to dark gray, pointed apically.

Thorax: anterior half fuscous suffused with blackish fuscous, posterior half grayish brown to fuscous, posterior crest distinct, dark brown. Collar gray. Tegula fuscous suffused with grayish brown. Hind tibia (Figure 11) in male not expanded, with a pale yellow hair pencil extending from the base along the inner surface. Forewing length 6.5–7.5 mm; elongate subrectangular, costa slightly curved, apex blunt, termen straight; upperside dark fuscous to blackish fuscous; costa with nine pairs of strigulae from the base to R_4_, which arranged in the same way as *S. miniscula*; strigulae one and two leaden, striae from them extending to basal half of dorsum, discontinuous; distal seven pairs of strigulae white; a large triangular mark tinged with ochreous centered on costa between 2/5 and 3/4 length, reaching halfway across the wing and bordered by a V‐shaped blackish fuscous band, which extends to the middle of dorsum; distal area intricately marked by broken leaden striae originating from costal strigulae; cilia fuscous; the underside is black brown and the overlapping part with the hindwing is leaden. Hindwing brown except pale yellow in area of overlap with forewing; cilia gray, with pale brown baseline; underside brown.

Abdomen: pale fuscous dorsally, pale yellowish‐brown ventrally. Male genitalia (Figure 15): tegumen narrow and high. Uncus broad, bilobed caudally, with long spines. Socius large, pendent, elongate rounded, with densely long spines. Gnathos with median prominence long, distal part narrowed, less than half width of basal part, furcated apically, inverted T‐shaped, with the branches as long as stem; lateral prominences (Figure 15a) broad, tongue‐like, naked. Valva is slender, nearly straight; sacculus ill‐defined, with sparse short spines, more or less constricted beyond the basal excavation, a small longitudinal lobe along the ventral margin between sacculus and cucullus, broad and very short; a few long spines on the inside of the lobe; cucullus with sparse spines, basal part slightly broader than the distal part. Caulis narrow, anellus a narrow ring, aedeagus moderate in width and length, cornuti absent. Female unknown.

Etymology. The specific name is derived from the Latin *calvus* (=hairless), referring to the naked lateral prominences of gnathos in the male genitalia.

### 3.5 *Statherotmantis longiuscula* Jing et Yu, sp. nov.

Type material. Holotype: ♂, China, Zhejiang Prov.: Mt. Jiulong, 28°19′ N, 118°59′ E, 623 m, 5.VII.2013, leg. Aihui Yin and Xiuchun Wang, genitalia slide no. JWQ21617. Paratype: Zhejiang Prov.: 3♂, 2♀, same data as holotype; 1♂, same data as holotype except 30°23′ N, 119°29′ E, 866 m, 13.VIII.2014; 1♂, Mt. Tianmu, 30°19′ N, 119°24′ E, 555 m, 17.VIII.2014, leg. Aihui Yin, Qingyun Wang and Suran Li; 1♀, Wangdongyang Reserve, 27°40′ N, 119°38′ E, 1301 m, 12.VIII.2016, leg. Qingyun Wang, Meiqing Yang and Ping Liu; Hubei Prov.: 1♀, Yingshan County, 30°59′ N, 116°01′ E, 635 m, 23.VI.2014, leg. Wei Guan and Meiqing Yang (Figures 4, 8, 12, 16 and 18).

This species is in GenBank as GenBank accession number: OR138302.1, OR138303.1.

Diagnosis. The male genitalia of *S. longiuscula* resembles a few congeners from China, such as *S. maoerica*, *S. expansa*, *S. spinulifera*, *S. calva* sp. n., and *S. triangularis*, but it can be distinguished by the bilobed uncus, the gnathos with distally furcated median prominence and short, naked lateral prominences, elongate valva with a slender basal lobe on the costa. Meanwhile, the median prominence of gnathos is rounded or truncate apicallly and the valva has no lobes on the costa in *S. maoerica* and *S. expansa*; the lateral promenences of gnathos are relatively large, tongue‐like in *S. spinulifera* and *S. calva* sp. n.; the uncus is rounded and the cucullus is elongate triangular in *S. triangularis*.

Description. Adult (Figure 4). Head (Figure 8): vertex roughly scaled, pale brown to pale gray. Frons pale brown to brown. Antenna brown to dark brown, reaching to the middle of the forewing. Labial palpus porrect or ascending, sinuate, creamy tinged with pale gray slightly, median segment distally moderately widened, terminal segment porrect, pointed.

Thorax: brown, suffused with fuscous medially and posteriorly. Collar brown. Tegula fuscous, dark brown distally. The hind tibia (Figure 12) is pale, with a short, pale yellow hair pencil extending from the base along the inner surface in males. Forewing length 7.0–8.0 mm; elongate subrectangular, costa slightly curved, apex blunt, termen straight; upperside fuscous to blackish fuscous; costa with nine pairs of strigulae from base to R_4_, which arranged in the same way as *S. miniscula*; basal two pairs leaden or pale fuscous, striae from them distinct, extending to basal half of dorsum, lower part discontinuous; distal seven pairs of costal strigulae white; a semicircular mark tinged with ochreous between 2/5 and 3/4 length of costa, reaching the upper edge of cell across wing, bordered by a significant blackish fuscous band, which zigzagging across wing from 1/3 length of costa to middle dorsum, then back towards apex, curving below the pair of costal strigula seven and divided into two branch, the proximal branch a straight band extending to end of dorsum, scattered with white scales, the distal branch ending in termen between R_5_ and M_3_, proximal edge with a narrow white line; cilia pale fuscous suffused with dark fuscous; underside is dark brown, the overlapping part with the hindwing is gray. Hindwing dark brown; cilia gray; underside dark brown.

Abdomen: pale brown dorsally, pale yellow ventrally. Male genitalia (Figure 16): tegumen high and narrow. Uncus broad, rounded, and bilobed caudally, with dense hairspines. Socius pendent, large and oval, with densely long spines. Gnathos with median prominence broad, distal half furcated, the branches narrow and away from each other; lateral prominences small rounded angle, short, and naked. Valva slender, roughly straight, with an elongate, naked costal prominence at the base (Figure 16a); sacculus sparsely setose, with a transversal lobe medially, somewhat triangular, with short spines; cucullus with sparse thorns, ventral edge sinuate, ventroproximal base slightly projecting, bearing one or two short thorns apically. Caulis is short and very thin, anellus a narrow ring, aedeagus slender, moderate in length, cornuti absent. Female genitalia (Figure 18): ovipositor lobes subtriangular. Apophyses anterior is short, about half the length of the apophyses posterior. The 8th tergite is well sclerotized. Sternum 7 with a weakly concave hindmargin. Sterigma derived from a raised fold encircling the ostium, with lateral portions produced posteriorly into spinulose points. Ostium rounded. Ductus bursae are long and smooth, with a bipartite colliculum below the ostium. Corpus bursae ovate, densely granulated, with two pectinate signa, almost the same size.

Etymology. The specific name is derived from the Latin *longiusculus* (=long), referring to the long median prominence of gnathos in the male genitalia.

### 3.6 Molecular data analyses

**FIGURE 19:**
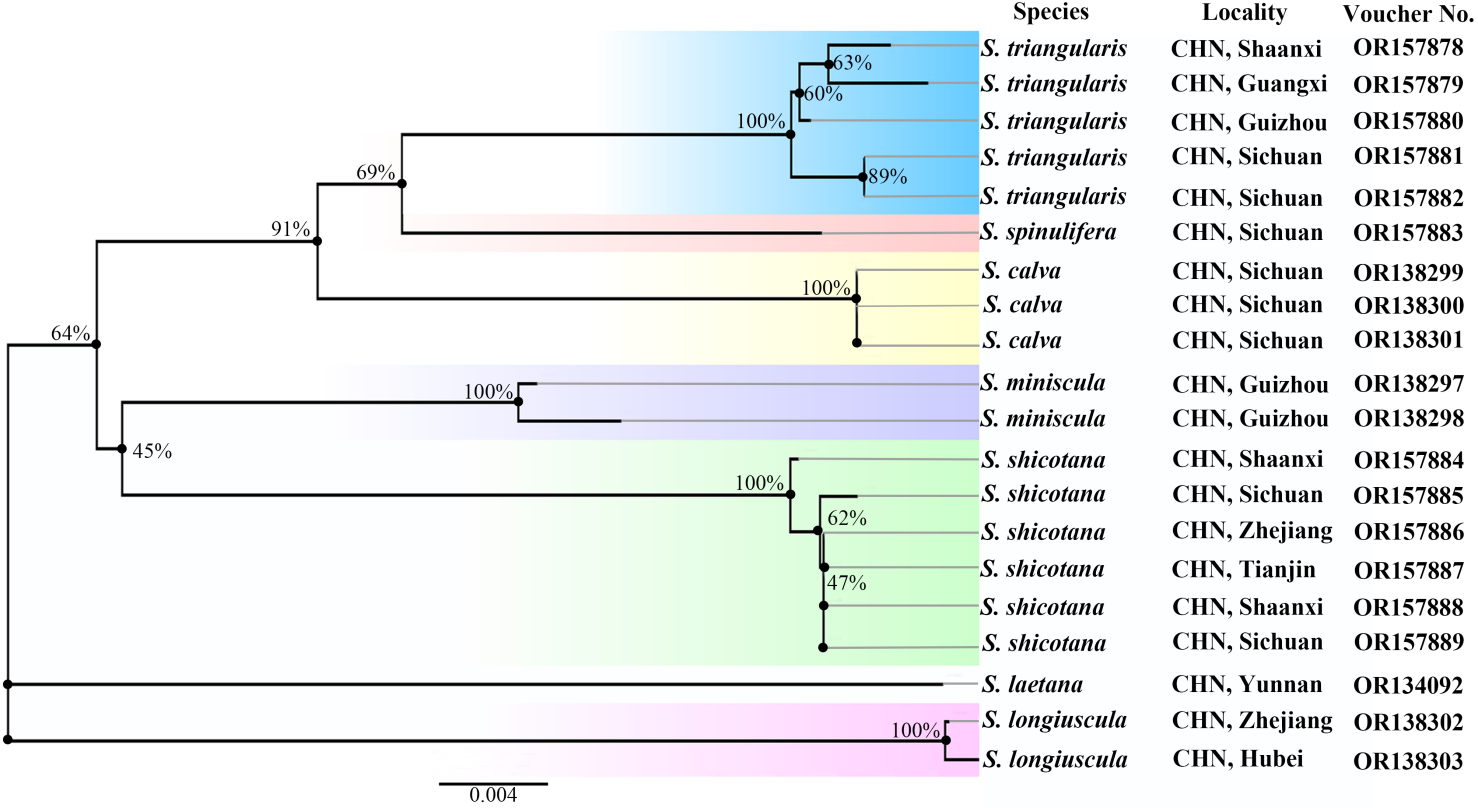
Neighbor‐joining Tree, based on DNA barcodes of *Statherotmantis* species. Numbers above/below branches refer to bootstrap proportions.

A total of the 20 exemplar specimens were successfully sequenced (Table S1), and all COI gene sequences were 658 bp in length. Based on the neighbor‐joining analysis of these 20 sequences (Figure 19), revealed the presence of seven well‐supported clades with high‐bootstrap values, each clade represented one species. The genetic distances are presented in Table S2. The interspecific genetic distances within the seven species varied from 3.4% to 7.5%, and the average divergence was 5.9%. The intraspecific divergence in the five species (except *S. laetana* and *S. spinulifera*) varied from 0% to 0.5% and the average divergence was 0.26%.

## 4 DISCUSSION

All members of the genus look superficially very similar and combined with some species having minor interspecific differences, this makes it difficult to identify species. For example, due to the similarities in external appearance and genitalia, we regarded the specimens of *S. calva* sp. nov. as *S. spinulifera* formerly, but DNA barcodes demonstrated that these specimens actually belonged to a new species, genetic divergence between these specimens and those of *S. spinulifera* is 4.2%, which exceeds the commonly accepted threshold of 2% for distinguishing different species in Lepidoptera (Hajibabaei et al., 2006; Zahiri et al., 2014); indeed, the observed variations in genitalia among the species can be considered as interspecific character variability, as mentioned earlier. These variations in the genitalia serve as important diagnostic features for species identification. The similar forewing patterns in related species also make it difficult to associate males and females. However, in this present study, there are no female specimens of different species collected from the same location. By focusing on male specimens and their corresponding DNA barcodes, the analysis and assessment of species diversity can be carried out with greater clarity and accuracy.

In Chinese fauna, distinguishing between *Statherotmantis* and *Statherotis discana* (Felder *et* Rogenhofer, 1875) as well as *Cephalophyes porphyrea* Diakonoff, 1973 base on forewing patterns along is almost impossible. However, there is a distinctive morphological characteristic that the males of the latter two species have an oval patch of blackish androconial scales on the lower surface of the discal cell in the forewing and central area of the upper surface in the hindwing respectively, which is absent in males of *Statherotmantis*. Therefore, when it comes to differentiating these species, it becomes crucial to examine the presence or absence of these specific androconial scale patches in male specimens. This additional morphological feature provides a reliable distinguishing characteristic that can aid in the accurate identification of *Statherotis discana*, *Cephalophyes porphyrea*, and *Statherotmantis* individuals in the Chinese fauna.

## AUTHOR CONTRIBUTIONS

**Wenqing Jing:** Conceptualization (lead); investigation (lead); software (lead); visualization (lead); writing – original draft (lead); writing – review and editing (equal). **Zehua Niu:** Conceptualization (supporting); methodology (supporting); software (supporting); writing – review and editing (equal). **Shuang Ma:** Conceptualization (supporting); validation (supporting). **Haili Yu:** Conceptualization (supporting); funding acquisition (lead); methodology (lead); validation (supporting); writing – review and editing (equal).

## FUNDING INFORMATION

The study was funded by the Foundation of the Shaanxi Educational Committee of China, grant no. 18JS107.

## CONFLICT OF INTEREST STATEMENT

None.

## Supporting information


Tables S1–S2:

## Data Availability

DNA sequence data is deposited on GenBank, accession numbers: OR157878.1–OR157889.1, OR134092.1, OR138297.1–OR138303.1.
