# Supplementary material for: New species and new record of Statherotmantis Diakonoff, 1973 from China (Lepidoptera: Tortricidae: Olethreutinae)
Source: Ecol Evol. 2024 Jan 31;14(2):e10906. doi: 10.1002/ece3.10906 (PMC10830350; doi:10.1002/ece3.10906)
Supplement: Supplementary file 1 — Tables S1–S2: [file ECE3-14-e10906-s001.doc]

**New species and new record of *Statherotmantis* Diakonoff, 1973 from China (Lepidoptera, Tortricidae, Olethreutinae)**

Wenqing Jing, Zehua Niu, Shuang Ma & Haili Yu

**Supplementary Data**

**Contents**

Supplementary Table 1: Species sampled for the molecular analysis . 2

Supplementary Table 2: Pairwise distances calculated within and between *Statherotmantis* species resulting from COI gene dataset . 3

Supplementary table 1. Species sampled for the molecular analysis.

| **Species** | **Locality** | **Data collected** | **GenBank aaccession number** | **Reference** |
| --- | --- | --- | --- | --- |
| *Statherotmantis triangularis* | China, Shaanxi Prov., Langao County (32°13' N, 108°48' E) | 13.VIII.2016 | OR157878.1 | present study |
| *S. triangularis* | China, Guangxi Prov., Mt. Mao’er (25°52' N, 110°29' E) | 25.VII.2015 | OR157879.1 | present study |
| *S. triangularis* | China, Guizhou Prov., Mt. Leigong (26°22' N, 108°12' E) | 01.VIII.2018 | OR157880.1 | present study |
| *S. triangularis* | China, Sichuan Prov., Baoxing County (30°34' N, 102°52' E) | 01.VIII.2016 | OR157881.1 | present study |
| *S. triangularis* | China, Sichuan Prov., Yingjing County (29°37' N, 102°52' E) | 16.VII.2021 | OR157882.1 | present study |
| *S. spinulifera* | China, Sichuan Prov., Mt. Siguniang (30°35' N, 102°30' E) | 06.VII.2016 | OR157883.1 | present study |
| *S. shicotana* | China, Shaanxi Prov., Zhouzhi County (33°50' N, 107°49' E) | 02.VI.2022 | OR157884.1 | present study |
| *S. shicotana* | China, Sichuan Prov., Baoxing County (30°34' N, 102°52' E) | 26.VII.2021 | OR157885.1 | present study |
| *S. shicotana* | China, Zhejiang Prov., Mt. Baizu (27°44' N, 119°10' E) | 17.VIII.2016 | OR157886.1 | present study |
| *S. shicotana* | China, Tianjin County, Mt. Baxian (40°6' N, 117°19' E) | 26.VII.2018 | OR157887.1 | present study |
| *S. shicotana* | China, Shaanxi Prov., Ningqiang County (32°44' N, 106°13' E) | 22.VII.2022 | OR157888.1 | present study |
| *S. shicotana* | China, Sichuan Prov., Baoxing County (30°34' N, 102°52' E) | 27.VII.2021 | OR157889.1 | present study |
| *S. laetana* | China, Yunnan Prov., Mt. Gaoligong (27°41' N, 98°16' E) | 1.VI.2017 | OR134092.1 | present study |
| *S. miniscula* sp. nov. | China, Guangxi Prov., Leishan County (26°22' N, 108°11' E) | 17.VI.2019 | OR138297.1 | present study |
| *S. miniscula* sp. nov. | China, Guangxi Prov., Mt. Leigong (26°22' N, 108°11' E) | 20.VI.2019 | OR138298.1 | present study |
| *S. calva* sp. nov. | China, Sichuan Prov., Mt. Emei (29°34' N, 103°24' E) | 20.VII.2021 | OR138299.1 | present study |
| *S. calva* sp. nov. | China, Sichuan Prov., Mt. Emeishan (29°34' N, 103°24' E) | 20.VII.2021 | OR138300.1 | present study |
| *S. calva* sp. nov. | China, Sichuan Prov., Mt. Emeishan (29°34' N, 103°24' E) | 20.VII.2021 | OR138301.1 | present study |
| *S. longiuscula* sp. nov. | China, Zhejiang Prov., Mt. Jiulong (28°19' N, 118°59' E) | 5.VII.2013 | OR138302.1 | present study |
| *S. longiuscula* sp. nov. | China, Hubei Prov., Yingshan County (30°59' N, 116°02' E) | 23.VI.2014 | OR138303.1 | present study |

Supplementary table 2. Pairwise distance calculated within and between *Statherotmantis* species resulting from COI gene dataset.

| **Species** | **GenBank accession number** | **Locality** | **1** | **2** | **3** | **4** | **5** | **6** | **7** | **8** | **9** | **10** |
| --- | --- | --- | --- | --- | --- | --- | --- | --- | --- | --- | --- | --- |
| *S. trangularis* | OR157878.1 | CHN, Shaanxi |  |  |  |  |  |  |  |  |  |  |
| *S. trangularis* | OR157879.1 | CHN, Guangxi | 0.006 |  |  |  |  |  |  |  |  |  |
| *S. trangularis* | OR157880.1 | CHN, Guizhou | 0.003 | 0.006 |  |  |  |  |  |  |  |  |
| *S. trangularis* | OR157881.1 | CHN, Sichuan | 0.006 | 0.009 | 0.003 |  |  |  |  |  |  |  |
| *S. trangularis* | OR157882.1 | CHN, Sichuan | 0.006 | 0.009 | 0.003 | 0.000 |  |  |  |  |  |  |
| *S. spinulifera* | OR157883.1 | CHN, Sichuan | 0.034 | 0.036 | 0.031 | 0.034 | 0.034 |  |  |  |  |  |
| *S. shicotana* | OR157884.1 | CHN, Shaanxi | 0.059 | 0.061 | 0.056 | 0.059 | 0.059 | 0.056 |  |  |  |  |
| *S. shicotana* | OR157885.1 | CHN, Sichuan | 0.063 | 0.064 | 0.059 | 0.059 | 0.059 | 0.059 | 0.003 |  |  |  |
| *S. shicotana* | OR157886.1 | CHN, Zhejiang | 0.061 | 0.063 | 0.057 | 0.061 | 0.061 | 0.057 | 0.002 | 0.002 |  |  |
| *S. shicotana* | OR157887.1 | CHN, Tianjin | 0.061 | 0.063 | 0.057 | 0.061 | 0.061 | 0.057 | 0.002 | 0.002 | 0.000 |  |
| *S. shicotana* | OR157888.1 | CHN, Shaanxi | 0.061 | 0.063 | 0.057 | 0.061 | 0.061 | 0.057 | 0.002 | 0.002 | 0.000 | 0.000 |
| *S. shicotana* | OR157889.1 | CHN, Sichuan | 0.061 | 0.063 | 0.057 | 0.061 | 0.061 | 0.057 | 0.002 | 0.002 | 0.000 | 0.000 |
| *S. laetana* | OR134092.1 | CHN, Yunnan | 0.071 | 0.071 | 0.067 | 0.066 | 0.066 | 0.067 | 0.067 | 0.069 | 0.069 | 0.069 |
| *S. miniscula* sp. nov. | OR138297.1 | CHN, Guizhou | 0.049 | 0.049 | 0.046 | 0.049 | 0.049 | 0.044 | 0.041 | 0.044 | 0.042 | 0.042 |
| *S. miniscula* sp. nov. | OR138298.1 | CHN, Guizhou | 0.051 | 0.051 | 0.047 | 0.051 | 0.051 | 0.049 | 0.046 | 0.049 | 0.047 | 0.047 |
| *S. calva* sp. nov. | OR138299.1 | CHN, Sichuan | 0.043 | 0.046 | 0.039 | 0.038 | 0.038 | 0.042 | 0.059 | 0.057 | 0.057 | 0.057 |
| *S. calva* sp. nov. | OR138300.1 | CHN, Sichuan | 0.043 | 0.046 | 0.039 | 0.038 | 0.038 | 0.042 | 0.059 | 0.057 | 0.057 | 0.057 |
| *S. calva* sp. nov. | OR138301.1 | CHN, Sichuan | 0.043 | 0.046 | 0.039 | 0.038 | 0.038 | 0.042 | 0.059 | 0.057 | 0.057 | 0.057 |
| *S. longiuscula* sp. nov. | OR138302.1 | CHN, Zhejiang | 0.069 | 0.068 | 0.066 | 0.069 | 0.069 | 0.066 | 0.069 | 0.071 | 0.069 | 0.069 |
| *S. longiuscula* sp. nov. | OR138303.1 | CHN, Hubei | 0.071 | 0.069 | 0.067 | 0.071 | 0.071 | 0.067 | 0.071 | 0.072 | 0.071 | 0.071 |

Supplementary table 2. (Continued).

| **Species** | **GenBank accession number** | **Locality** | **11** | **12** | **13** | **14** | **15** | **16** | **17** | **18** | **19** |
| --- | --- | --- | --- | --- | --- | --- | --- | --- | --- | --- | --- |
| *S. shicotana* | OR157889.1 | CHN, Sichuan | 0.000 |  |  |  |  |  |  |  |  |
| *S. laetana* | OR134092.1 | CHN, Yunnan | 0.069 | 0.069 |  |  |  |  |  |  |  |
| *S. miniscula* sp. nov. | OR138297.1 | CHN, Guizhou | 0.042 | 0.042 | 0.060 |  |  |  |  |  |  |
| *S. miniscula* sp. nov. | OR138298.1 | CHN, Guizhou | 0.047 | 0.047 | 0.062 | 0.005 |  |  |  |  |  |
| *S. calva* sp. nov. | OR138299.1 | CHN, Sichuan | 0.057 | 0.057 | 0.073 | 0.044 | 0.049 |  |  |  |  |
| *S. calva* sp. nov. | OR138300.1 | CHN, Sichuan | 0.057 | 0.057 | 0.073 | 0.044 | 0.049 | 0.000 |  |  |  |
| *S. calva* sp. nov. | OR138301.1 | CHN, Sichuan | 0.057 | 0.057 | 0.073 | 0.044 | 0.049 | 0.000 | 0.000 |  |  |
| *S. longiuscula* sp. nov. | OR138302.1 | CHN, Zhejiang | 0.069 | 0.069 | 0.074 | 0.059 | 0.061 | 0.074 | 0.074 | 0.074 |  |
| *S. longiuscula* sp. nov. | OR138303.1 | CHN, Hubei | 0.071 | 0.071 | 0.076 | 0.057 | 0.059 | 0.076 | 0.076 | 0.076 | 0.002 |
